# Force-based three-dimensional model predicts mechanical drivers of cell sorting

**DOI:** 10.1098/rspb.2018.2495

**Published:** 2019-01-23

**Authors:** Christopher Revell, Raphael Blumenfeld, Kevin J. Chalut

**Affiliations:** 1 Cavendish Laboratory, Department of Physics, University of Cambridge, Cambridge CB3 0HE, UK; 2 Wellcome Trust/Medical Research Council Stem Cell Institute, University of Cambridge, Cambridge CB2 1QR, UK; 3 Department of Earth Science and Engineering, Imperial College London, London SW7 2BP, UK

**Keywords:** differential interfacial tension, self-organization, cortical tension, cell sorting, biological physics, modelling

## Abstract

Many biological processes, including tissue morphogenesis, are driven by cell sorting. However, the primary mechanical drivers of sorting in multicellular aggregates (MCAs) remain controversial, in part because there is no appropriate computational model to probe mechanical interactions between cells. To address this important issue, we developed a three-dimensional, local force-based simulation based on the subcellular element method. In our method, cells are modelled as collections of locally interacting force-bearing elements. We use the method to investigate the effects of tension and cell–cell adhesion on MCA sorting. We predict a minimum level of adhesion to produce inside-out sorting of two cell types, which is in excellent agreement with observations in several developmental systems. We also predict the level of tension asymmetry needed for robust sorting. The generality and flexibility of the method make it applicable to tissue self-organization in a myriad of other biological processes, such as tumorigenesis and embryogenesis.

## 1. Introduction

Self-organization is a widely studied feature of non-equilibrium systems across a great variety of fields [1–3]. In biology, self-organized processes range in length and time scales from protein folding [4] to the dramatic murmurations of starlings [5]. Generically, these systems exhibit emergence of macroscopic order from disordered states by the action of simple, local, inter-component interactions. Here, we focus on one such process of self-organization: cell sorting. Cell sorting in a multicellular aggregate (MCA) is critical for normal development of an embryo, and is also at the heart of pathologies such as metastatic growth of tumours.

The spontaneous separation of mixtures of embryonic cells has long been thought to be driven by cells’ differing ‘affinity’ for one another [6]. Two primary drivers have been proposed to facilitate differential affinity: differential adhesion and differential interfacial tension.

The differential adhesion hypothesis is a widely studied explanation of cell sorting [7–9]. It asserts that, in an MCA with multiple cell types, those with strong mutual adhesion are drawn together better than cells with weaker mutual adhesion. The latter are thus ‘sorted’ to the outside of the MCA. Although the differential adhesion hypothesis has been applied to a number of systems [10], it has been challenged in the light of observations that sorting occurs even when two cell types adhere to one another with equal strength [11], and that contractile forces are more significant than adhesion for tissue morphogenesis in a number of systems [12,13].

The idea that sorting in many biological processes is driven primarily by contractile forces is at the heart of the differential interfacial tension hypothesis [14], the basic postulates of which are: (i) cell surface membranes exhibit local tension variation [15]; (ii) a cell's surface tension is highest when in contact with the external medium and lowest when in contact with cells of the same type [16]. The variation in surface tension at cell interfaces leads to variation in the cell interface area, and hence to mutual affinity (figure 2*a*). According to the differential interfacial tension hypothesis, variations in mutual affinity, driven by differences in cortical tension, are primarily responsible for self-organization in a cell aggregate. The reduction in tension at cell interfaces is proposed to occur by the exclusion of myosin from the actomyosin cortex at the interface. Such exclusion is caused by the intra-membrane proteins that mediate adhesion between the cells [13]. This passive mechanism allows morphogenesis to proceed without active movements by the cells involved and, moreover, provides the local coordination between cells needed for complex global morphogenesis [17].

The importance of intercellular differential interfacial tension for tissue morphogenesis and maintenance has been demonstrated in a number of systems. For example, it is responsible for boundary maintenance in the dorsoventral compartment boundary of the *Drosophila* wing imaginal disc [18,19]. Much of this body of work has focused on two-dimensional epithelial systems, often maintaining boundaries rather than forming boundaries from a mixed aggregate [20]. However, further evidence of the importance of differential interfacial tension comes from experimental work on three-dimensional aggregates, suggesting that local variations in cortical tension are responsible for internalizing the first set of internal cells in the mouse morula [21]. Moreover, reduction in interfacial tension has also been shown to drive morula compaction [22] and allocation of cells to the inner cell mass of the embryo [23].

In order to investigate in detail the effect of differential interfacial tension on three-dimensional MCAs, we constructed a computational model based on the subcellular element method (SCEM) [24]. To validate the method, we compared its predictions to theoretical models of differential interfacial tension in cell doublets [13] (figure 2*c*). Using our model to simulate sorting in three-dimensional MCAs of two cell types, we were able to predict the minimum adhesion magnitude required for tension-driven sorting to occur. We show that, given this minimum adhesion, interfacial tension asymmetries drive sorting in MCAs. We also show that fairly significant differential adhesion is necessary for cell sorting. Ultimately, we also quantitatively predict the amount of tension asymmetry necessary for sorting.

## 2. Model

A number of techniques have been developed to model cellular systems [25–28], including explorations of differential interfacial tension using the vertex method in two-dimensional epithelia [19,29]. However, many of these methods rely on a global Hamiltonian, which is a strong, abstract assumption for modelling biological systems, which do not necessarily conserve energy. Instead, we set out to develop a computational model that assumes dynamics driven by local forces. We required a method that allows integration of subcellular-scale mechanics, including stiffness, tension and adhesion, with three-dimensional tissue-scale phenomena. Therefore, we chose to implement a model based on the SCEM.

**Figure 1.**
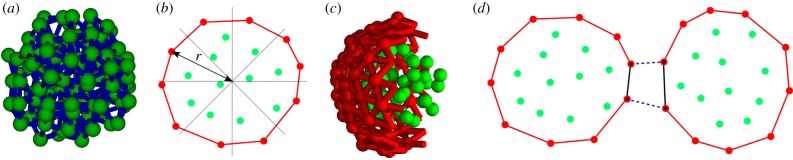
(*a*) Visualization of one SCEM cell showing elements as green spheres and nearest-neighbour forces as blue cylinders. (*b*) Algorithm for allocation of cortex elements, two-dimensional representation. The cell is divided into *π*/4 radian bins. Elements with a radius greater than 80% of the maximum radius in their bin are allocated cortex type. (*c*) Cutaway of one SCEM cell showing all cytoplasm elements and part of the cortical tension network as defined by a Delaunay triangulation. (*d*) Algorithm for implementing differential interfacial tension between two cells. Solid lines indicate cortical tension forces; dotted lines indicate inter-cell adhesive interactions. Elements that share adhesive interactions with another cell are labelled according to the type of cell, shown with a black dot. An interface is defined as a region in which cortex elements have the same label. The tension is altered for any cortex interaction between two cortex elements with the same label, shown by solid black lines. Cytoplasm elements are green and cortex elements are red throughout.

SCEM is a method for multi-scale three-dimensional simulation that can simultaneously model intra- and inter-cellular dynamics, as well as the development of multicellular tissues. It allows us to study the effects of cell shape changes and complex inter- and intra-cell features on tissue-scale dynamics [30]. The basic SCEM method [24] treats each cell as a collection of elements (figure 1*a*) interacting via nearest-neighbour Morse potentials of the form $V(r)=D_{e}({\text{e}}^{-2a(r-r_{e})}-2{\text{e}}^{-a(r-r_{e})}+1)$ [31]. Further details, including routines for cell growth and division, are discussed in electronic supplementary material, appendix A.

Nearest-neighbour elements of different cells also interact, facilitating intercellular interactions by the same mechanism. Different element types, however, can have different potential parameters. Element positions are updated at each time step according to these Morse potential interactions using overdamped Langevin dynamics: $\gamma \dot{\underline{x}}=-\nabla U(\underline{x})+\underline{\zeta }$.

For the purposes of this work, we modified the original SCEM so that each cell possesses a contractile cortex, the properties of which are different from the main bulk of the cell. With this contractile cortex, we can independently control intercellular adhesion, cortical tension and interfacial tension. This modification involved first identifying the surface elements of the cell. This was done by dividing the cell into 32 sectors of equal steradians, emanating from its centre of mass, and defining as cortex type all elements in a sector with a radius greater than 80% that of the furthest element in that sector (figure 1*b*). The allocation of cortex elements is updated at each time step to accommodate element movement or creation. Cortex elements that move to the centre of the cell are reallocated cytoplasm type, and cytoplasm elements that move to the boundary are allocated cortex type. Once the cortex elements are defined, inter-cellular adhesive interactions are mediated exclusively between cortex elements in different cells. Cytoplasm elements have only repulsive interactions with elements in different cells, which prevents cells from overlapping in space.

To ensure that the interaction between cortex elements is tangential to the cell surface, we performed a Delaunay triangulation [32,33] over each cell’s cortex elements, which makes the cell a polyhedron of triangular facets. By its nature, this triangulation favours roughly equilateral triangles, which produces a relatively smooth cell surface (figure 1*c*; electronic supplementary material, appendix C). Two cortex elements sharing such a triangle edge are considered neighbours and they interact along the edge via a tension force. In cellular systems, this is an active pulling force driven by actomyosin contraction, and thus is treated as a force of constant magnitude that does not vary with element separation. The equilibrium separation of elements is defined by a sum of the bulk interaction forces between elements and the additional tension component. Thus defined, the cortex forces are guaranteed to span the full cell surface, be tangential to it, and enable the modelling of intercellular mechanical interactions. The Delaunay triangulation also prevents elements from detaching from the cell; any cytoplasm element that escapes will immediately be allocated cortex type and experience a returning force.

With our computational method, the cortical tension can vary locally across the cell surface in response to contact with surfaces of other cells. Such variation is achieved by changing the magnitude of intra-cell tension forces between cortex elements that lie at a cell interface. Specifically, we define a cell interface by first identifying the cortex elements that share an adhesive inter-cell interaction with a cortex element of a different cell. These elements are then labelled according to whether the other cell is of the same or different type. An interface between the two cells is defined as a region containing only cortex elements of the same label, and any cortical tension force acting between two cortex elements with the same label can be altered (figure 1*d*). The magnitude of interfacial tension can be specified differently depending on cell type and whether the interface is between like or unlike cell types. Although the factor by which tension is updated for a given interface depends upon the types of cells at the interface, the same factor is applied to interfacial tension in both of the two cells at the interface.

Changing the local interaction between cell cortex elements affects not only the local cortical tension but also the distance between the elements and, therefore, the local element density. Since intercellular adhesion is mediated by these elements, an increase in element density, for example, increases the local adhesion strength between neighbouring cells. To compensate for this, we normalize the adhesion magnitude by the local element density (electronic supplementary material, appendix D). Parameters controlling system behaviour in our simulations are outlined in electronic supplementary material, appendix B.

## 3. Simulation of cell doublets

The simplest experimental system to define the mechanical phenotype of cells and their interfaces is a cell doublet, for which *in vitro* experiments and theoretical models exist. This makes this system a suitable test case to validate our method. We expect sorting to be driven by changes in relative affinity, reflected by changes in equilibrium interfacial contact area (or, analogously, contact angle, which is more tractable to measure experimentally) between cells. This interfacial contact area depends upon the adhesion magnitude between cells (*A*_M_) the cortical tension of the cells (*γ*_*m*_) and the interfacial tension (*γ*_*c*_).

**Figure 2.**
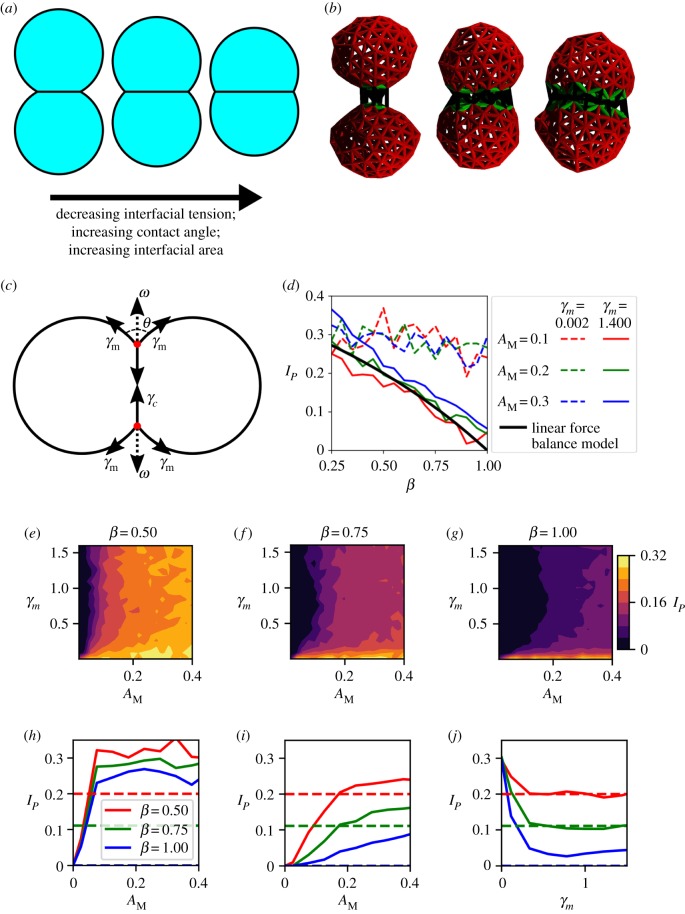
(*a*) Two-dimensional diagram of cell doublets showing the effect of changing interfacial tension on contact angle and interface area. Interfacial tension decreases from left to right, resulting in increased interfacial area, increased contact angle and consequently increased mutual affinity between cells. (*b*) Cell doublets visualized by cortical tension force network, demonstrating variation of interface area for interfacial tension factor values *β* = 1.00, 0.75, 0.50. (*c*) Diagram of linear force balance predicting cell doublet contact angle, as presented in [13]. Cylindrical symmetry allows the system to be reduced to a two-dimensional force balance at the vertices of the cell interface. The influence of adhesion (*A*_M_) is reframed as a colinear tensional force *ω* acting to pull the vertices apart. In order to reach equilibrium, the forces pulling the edges apart must balance the forces pulling the edges together. (*d*) Plots of interface proportion against *β* in high-tension and low-tension regimes. (*e*–*g*) Phase diagrams showing variation of interface proportion (*I*_*P*_) in *γ*_*m*_ and *A*_M_ space measured in cell doublet simulations. (*e*) *β* = 0.5. (*f*) *β* = 0.75. (*g*) *β* = 1.0. Force balance predicts *I*_*P*_ = 0.20, 0.11, 0.00, respectively. (*h*–*j*) Plots of interface area against adhesion and tension magnitude. Dotted lines show predictions of LFB. Colour coding of lines is the same for all three plots. (*h*) Interface against adhesion in low-tension regime, *γ*_*m*_ = 0.01. (*i*) Interface against adhesion in high-tension regime, *γ*_*m*_ = 1.2. (*j*) Interface against tension magnitude at *A*_M_ = 0.2.

Assuming a cell doublet is in mechanical equilibrium and has cylindrical symmetry, the dependence of the contact angle between two cells on their surface tensions can be deduced using linear force balance at the vertex [13] (figure 2*c*). Further assuming, based on experimental observations [13,34,35], that the effect of adhesion is negligible in the high-tension limit, one can derive a relationship between *γ*_*m*_, *γ*_*c*_ and the doublet contact angle *θ*: β=cos⁡(θ/2) where $\beta =\gamma_{c}/\gamma_{m}$. From here, we call this model, which neglects adhesion and assumes that cell–cell affinity reflects a balance of cortical and interfacial tension, the linear force balance model.

To test the effect of system parameters on cell doublets in our simulations, we allow an initial cell to divide into two, with both daughter cells having the same properties, and specify a corresponding value of *β* for the interface formed between them. We then allow the system to reach mechanical equilibrium without growth or division, producing a doublet of identical cells, adhered at a shared interface (figure 2*b*). An alternative protocol—bringing two cells into contact and allowing an interface to form from nothing—was assessed to be less representative of behaviour in real MCAs.

Experimentally, a straightforward indicator of cell affinity in doublets is the contact angle between the two cells in a doublet, *θ*. While this measurement is intractable in our simulations, we could straightforwardly measure the cell–cell interface area as the sum of the areas of all the Delaunay triangles whose vertex elements all interact with elements in the neighbouring cell, and express this area as the ratio, *I*_*P*_ of the interface area to the total cell surface area. Using simple trigonometry to relate interfacial area to contact angle, *θ*, we found the corresponding prediction of the linear force balance model for *I*_*P*_: *I*_*P*_ = (1 − *β*)/(3 − *β*). This allows direct comparison of our simulations to the linear force balance model at cell interfaces and provides a validation test of our method’s predictions (figure 2*d*). The value of *θ* can also be measured in experiments. The validation consisted of simulating cell doublets, from which we obtained measurements of *I*_*P*_ for values of *β* between 0.25 and 1. We define an adhesion magnitude *A*_M_ for our simulations. The value of *A*_M_ is the maximum force applied between elements across the interaction range of the adhesive potential. For these doublet simulations, we used a representative set of *A*_M_ and *γ*_*m*_ values, corresponding to low-tension and high-tension regimes. The resulting *I*_*P*_ values were then compared to the theoretical predictions of the linear force balance model (figure 2*d*).

We found very good agreement between the simulation results and the theoretical prediction of the force balance model in the high-tension regime (solid lines in figure 2*d*), with adhesion producing a small constant offset across *β*. The magnitude of this offset increases gradually with adhesion. By contrast, in the low-tension regime, adhesion cannot be ignored when considering the linear force balance in a cell doublet. Moreover, two cells with no adhesion cannot form an interface, and thus the linear force balance model is unsurprisingly not appropriate to use for low-adhesion conditions. Indeed, the simulation predicts a sharp drop in the interface at low adhesion. Most importantly, we conclude that due to the excellent agreement with the linear force balance model in the high-tension regime, our method can be used to predict the mechanical interactions within MCAs.

By simulating a large number of cell doublets at different parameters, we constructed phase diagrams for the behaviour of the cell doublet interface area as a function of *A*_M_ and *γ*_*m*_ for *β* = 0.5, 0.75 and 1.00 (figure 2*e*–*g*). The phase diagrams indicate that the highest *I*_*P*_ value achieved for any parameter set is approximately 0.32. This value is in good agreement with the theoretical limit for the interface between two hemispheres, which is exactly 1/3. Our doublet simulations also show that, for each value of *β*, the measurement of *I*_*P*_ drops sharply with increasing *γ*_*m*_, tending to a minimum (figure 2*j*). The minimum is determined by *A*_M_, with very low adhesions producing little to no interface. Increasing adhesion from zero produces a sharp increase in the interface, but beyond a threshold adhesion the increase of interface with adhesion magnitude has a much smaller gradient (figure 2*h*).

The doublet simulation results are even clearer if we take slices through the phase diagram (figure 2*h*–*j*). As expected, we find that, for both low and high tensions, little to no interface is produced at low adhesion values. However, for both low and high tension, the interface increases approximately linearly with increasing adhesion. In the low-tension regime (figure 2*h*), interfacial contact area increases sharply with adhesion until a value of approximately *A*_M_ = 0.1, after which the interfacial contact area plateaus. The value at which the interfacial contact area plateaus is approximately equal to the theoretical limit for two hemispheres (0.33) at low *β*. In the high-tension regime (figure 2*i*), we observe two adhesion regimes. Below a value of *A*_M_ = 0.2, the interfacial contact area increases slowly with adhesion; beyond that value, the interfacial contact area depends very weakly on adhesion.

Our doublet simulations demonstrate that our method is an accurate predictor of the mechanical interactions between cells. Furthermore, differences between simulation results and theoretical predictions further highlight the constraints of using the linear force balance model in situations for which adhesion cannot be neglected. Importantly, our method does not require the low adhesion assumption and can be used in any limit of adhesion or tension.

## 4. Quantitative predictions of sorting in multicellular aggregates

Having validated our method and used it to study cell doublet mechanics, we now demonstrate the influence of adhesion and interfacial tension on the sorting of two cell types in MCAs.

In order to assess quantitatively the effect of mechanics on the extent and speed of sorting in a cell aggregate, we developed three measures of sorting. These measures of sorting, as described below, are continuously calculated during the evolution of an MCA, and compared to a randomized reference distribution to produce a sorting index.

(1) Radius measure. We can consider sorting in spherical MCAs, as studied here, to be the aggregation of one cell type within the MCA. This can be quantified by taking the mean radius of all cells of that type from the centre of mass of that cell type. This quantity is our first sorting measure, referred to as the radius measure, *X*_*r*_.
(2) Neighbour measure. In a sorted system, we expect a higher number of same-type cell neighbours. We consider two cells to be neighbours if at least one element in one cell interacts with an element of the other. It is via such interactions that neighbour cells apply forces to one another, but the cells are counted as neighbours even if the magnitude of this force is zero. The neighbour measure, *X*_*n*_, is the total number of such like–like neighbour pairs of the cell type expected to sort to the centre of the aggregate.
(3) Surface measure. For spherical inside–outside sorting, we expect one cell type to occupy a greater proportion of the external surface of the MCA than the other. With our model, we are able to calculate the total cell surface area across all cells that is not part of a cell–cell interface. Therefore, for the surface measure, we calculate the fraction, *X*_*s*_, of the total cell surface area not in contact with other cells that is contributed by the type expected to sort to the outside.

**Figure 3.**
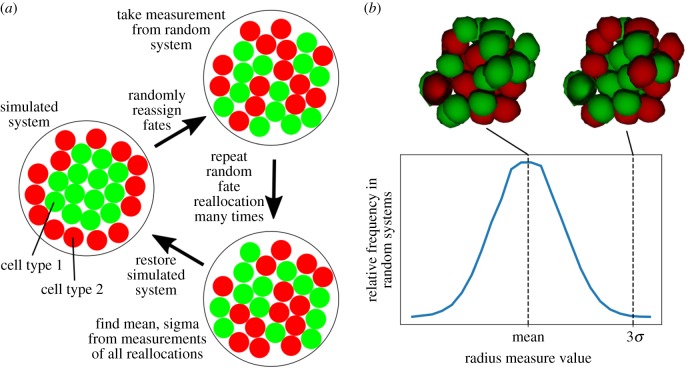
(*a*) Diagram showing how system states are reassigned to produce mean, standard deviation, maximum and minimum values of each sorting measure for a given spatial configuration and cell type ratio. Starting from the simulation state, shown on the left, the type of each cell is randomly reassigned, maintaining the same spatial distribution of cells and the same ratio of cell types. All sorting measures are calculated for this new state. Cell types are randomly reallocated 10^4^ times (electronic supplementary material, appendix E) to find the mean, maximum, minimum and standard deviation of sorting measures, before returning the system to the original simulation state. (*b*) Distribution of radius sorting measure values over random state reallocations of 30 cells found to be approximately Gaussian, allowing calculation of mean and standard deviation. The same pattern is seen for other sorting measures. Visualizations showing example system configurations at mean and 3*σ* shown above plot.

To contextualize the measures described above, we introduced uniformly randomized control systems, against which the measures could be compared. To generate such controls, the type of all cells in a system was randomly reassigned at each data output interval, while maintaining the same number of each cell type and the spatial configuration of cells, at which point the sorting measures were calculated again for these randomized systems (figure 3*a*). Repeating this procedure 10^4^ times (electronic supplementary material, appendix E) produces a normal distribution of the sorting measures (figure 3*b*) from which we can calculate the mean, *E*(*X*_*i*_), maximum, *X*_*i*,max_, minimum, *X*_*i*,min_ and standard deviation, *σ*(*X*_*i*_), of each sorting measure *X*_*i*_. After these repeats, the MCA is returned to its original state and the simulation continues. The sorting measure as calculated from the simulation, *X*_*i*_, is compared to the distribution of randomized measure values by calculating a sorting index, *S*_*i*_: *S*_*i*_ = (*X*_*i*_ − *E*(*X*_*i*_))/3*σ*(*X*_*i*_). Using *σ* in the divisor rather than the full range of randomized system values ensures that highly deviant results in the randomized distribution do not overly affect the sorting index. Defined in this way, we expect the sorting indices to run roughly from 0 to 1, with values near 0 indicating a randomly mixed system, and values near 1 indicating a sorted system.

For all following sorting simulations, we used our method to simulate MCAs growing from 10 to 30 cells with two different cell types. Once the system reached 30 cells, simulations were stopped and the final state of the system at that point was analysed. We define cell type 1 to be that expected to sort to the inside of the MCA, and cell type 2 to be that expected to sort to the outside (figure 3*a*). We calculated the radius, neighbour and surface sorting measures, and hence the respective sorting indices, at regular intervals. We can define three interfacial tension factors: like–like interfaces between both cell types (*β*_1,1_, *β*_2,2_) and unlike interfaces (*β*_1,2_). For simplicity in all simulations, we set *β*_1,2_ = *β*_2,2_ = 1.0 and vary *β*_1,1_, henceforth simply referred to as *β*. Therefore, cell type 1 is the reference cell type for calculating the sorting indices. Both cell types were given the same cortical tension. Furthermore, we restricted ourselves to the high cortical tension limit, and define the dimensionless adhesion parameter *α* = *A*_M_/*γ*_*m*_ to simulate the dynamics of MCAs for a wide range of values of *α* and *β*. We also assume, unless otherwise stated, the same adhesion magnitude for both cell types, for both like and unlike adhesion. Experimentally, *α* is difficult to measure, but it is thought to be in the range of 0.2–0.25 at the most [12,13,36], justifying our use of the high-tension regime as the biologically relevant regime.

To test also the effect of a division bias, we ran two types of simulations for each pair of *α* and *β* values: (i) a symmetric division, in which in which each division produced cells of the same type as the parent; (ii) an asymmetric division, each dividing cell had a 50% change of producing two daughter cells of the same type as the parent, and 50% chance of producing two daughter cells of different types. Each parameter set was repeated four times in order to estimate variability in the simulations.

We first used our method to test the hypothesis that doublet mechanics is a good predictor of sorting in an MCA, and then went on to use it to establish a better understanding of the mechanical drivers of cell sorting.

### (a) Doublet mechanics predicts sorting in multicellular aggregates

One of the main issues impeding the experimental validation of the mechanical drivers of sorting in MCAs is the difficulty of measuring forces within an MCA. Therefore, cell doublets are often used to quantify the forces that can be used to predict sorting in an MCA. We thus sought to test the hypothesis that cell doublets are a good and sufficient model for MCAs.

**Figure 4.**
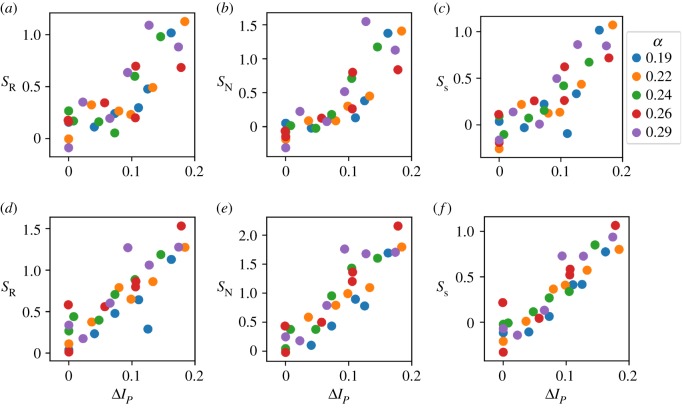
Plots of final sorting index for 30 cell aggregates against the difference in interface proportions for the two cell types found in doublet testing at corresponding parameter values. A strong relationship is seen between the difference in doublet interface proportion and final sorting index. Colour of point indicates adhesion (*α*). (*a*–*c*) Asymmetric division. (*d*–*f*) Symmetric division. (*a*,*d*) Radius measure. (*b*,*e*) Neighbour measure. (*c*,*f*) Surface measure. Values used for simulations: *α* = 0.19, 0.22, 0.24, 0.26, 0.29, *β* = 0.5, 0.6, 0.7, 0.8, 0.9, 1.0.

Based on ranges of published results, we ran the simulations for several random values *β* less than or equal to 1.0 and *α* less than or equal to 0.4 for both symmetric and asymmetric division. We quantified the sorting index for the final state of 30 different systems. We compared these values with the results of doublet testing (figure 2) with the same parameters. In this way, we can assess how measurements taken from cell doublets predict the extent of sorting in MCAs. Figure 4 shows final sorting indices plotted against the difference in doublet interface proportion (Δ*I*_*P*_). This difference is measured for like–like doublets of two cell types with the same parameters as the corresponding MCA. So, if the parameters used in the MCA simulation correspond to like–like doublet interface proportions of *I*_*P*,11_ for cell type 1 and *I*_*P*,22_ for cell type 2, where cell type 1 is expected to sort to the inside, Δ*I*_*P*_ = *I*_*P*,11_ − *I*_*P*,22_ for this MCA. The resulting scatter plots clearly show a nearly perfect positive correlation between the difference in interface proportion and the final sorting index across all sorting measures. This correlation is especially strong for systems with symmetric division. Importantly, this strong correlation establishes that experimentally tractable cell–cell affinity in a doublet, regardless of the underlying forces giving rise to that affinity, can be used to accurately predict the degree of sorting of MCAs. It also acts as further validation of the precision of our method in sorting MCAs.

### (b) Mechanical drivers of cell sorting in multicellular aggregates

**Figure 5.**
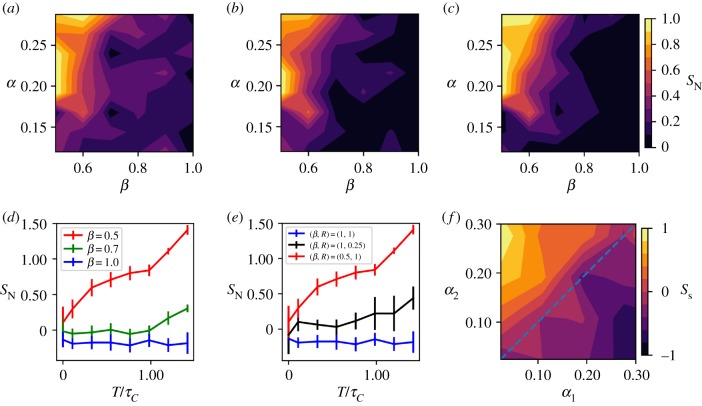
(*a*–*c*) Phase diagrams of final sorting index for 30 cell systems in *α* and *β* space using (*a*) radius measure, (*b*) surface measure and (*c*) neighbour measure. All cells have the same *γ*_*m*_, both cell types adhere with the same magnitude *α*, and all runs have asymmetric division. *β* refers to the interfacial tension factor of cell type 1. (*d*) Neighbour sorting index against *time*/*cell* *cycle* *time* (*T*/*τ*_*C*_) in aggregates with asymmetric division and *α* = 0.2. (*e*) Comparison of sorting kinetics for differential adhesion and differential interfacial tension where *R* is the adhesion ratio. (*f*) Example of sorting by differential adhesion with asymmetric division from surface measure.

In the following, we will use our simulations to make quantitative predictions about what adhesion and tension asymmetries are necessary for MCAs to sort. We performed simulations over a wide range of *α* and *β* values, and constructed a phase diagram in the *α* − *β* parameter space from the final value of sorting indices calculated at the end of each simulation (figure 5*a*–*c*). From these phase diagrams, we can deduce the limits on the values of *α* and *β* that drive sorting.

Figure 5*a*–*c* demonstrates that there is good agreement between sorting indices. Moreover, the extent of sorting increases as *β* is reduced. This is predictable, given that a reduction of *β* is indicative of a relaxation of interfacial tension at the expense of surface cortical tension for cell type 1. Significantly, with our method, we can now quantitatively predict the maximum of *β* (≈0.6) necessary to drive sorting at experimentally realistic values of *α*. We also see in figure 5*d* that the kinetics of sorting are faster for smaller values of *β*, with very little sorting occurring for *β* = 0.7. It is also clear that a minimum adhesion is required in order for the *β* parameter to have any effect: for values of *α* < 0.2, little to no sorting is observed regardless of the value of *β*. This is interesting because *α* = 0.2 is in agreement with experimental observations showing adhesion magnitude being around 0.2 times cortical tension magnitude in systems where cortical tension has been proposed as a significant factor in morphogenesis [12,13].

We also use our method to explore the effects of differential adhesion. As seen in (figure 5*f*) differential adhesion can drive sorting, but requires a large difference in adhesion magnitude between the cell types—approximately a factor of 10 for full sorting. We also see in figure 5*e* that sorting by differential adhesion with *A*_M,2_/*A*_M,1_ = *R* = 0.25 is slower than that driven by differential interfacial tension with *β* = 0.5. This suggests, in conjunction with the fact that adhesion forces are much smaller than tension forces, that tension asymmetry is a more significant factor in MCA sorting than adhesion asymmetry.

## 5. Discussion

We introduced a simulation method, an extension of SCEM, to investigate the mechanics of cell sorting. The model is based on local intra- and inter-cellular forces, and includes cell growth and division, and therefore evolution of different size MCAs. It also allows us to define different regions of a cell and the mechanical properties of those regions. Therefore, our method is highly flexible and can model intercellular and intracellular mechanical interactions in a broad range of multicellular systems.

We first employed the experimentally tractable cell doublet system both to validate our numerical model and to probe the effect of adhesion and tension on the interfacial contact area between two cells. In a doublet, this contact area is determined by the balance of the adhesion force, cortical tension, and interfacial tension. We studied the effects of these parameters on doublet interfacial contact area and found that it decreases sharply with interfacial tension before reaching a stationary phase in the high-tension regime. We also found that no interface can form when the adhesion drops below a threshold, above which the interfacial contact area depends only weakly on adhesion. Informed by experimental observations that tension forces are at least four times stronger than adhesion [12,13], we studied the high-tension regime and found that the variation of interfacial contact area with the tension parameter, *β*, agrees remarkably well with the predictions of using local force balance at cell interfaces, neglecting adhesion. This validates our method, showing that it captures important aspects of the underlying mechanics.

Next, we simulated the evolution of MCAs, growing from 10 to 30 cells, to study the effects of adhesion and tension on the sorting process. Using the novel, unbiased sorting index that we developed, we observed a very strong correlation between the sorting indices in the MCAs and the interfacial contact area between cells in doublets. This observation is significant because, while experimental determination of mechanics within MCAs is difficult, measurements of cortical tension and contact angle in cell doublets are relatively straightforward. Perhaps just as importantly, this observation confirms that even though interfacial tension, cortical tension and adhesion can all affect cell–cell affinity, it is only the combination of their contributions in the form of cell–cell affinity that affects cell sorting. Furthermore, these plots (figure 4*a*–*c*) suggest that a relatively large difference in doublet interface area between the two cell types is required to produce complete sorting reliably.

Finally, in order to disentangle the relative effects of differential interfacial tension and differential adhesion, we constructed phase diagrams for the sorting indices as a function of *α* and *β*. These phase diagrams show that DIT alone is sufficient to produce full sorting of MCAs with a sufficiently small *β*. Importantly, measurements of this experimentally accessible parameter in cell doublets can be used as a test of this prediction. Furthermore, we found that there is a lower bound for adhesion necessary for sorting of an MCA which is in agreement with experimentally observed values. In contrast to differential interfacial tension, the amount of differential adhesion necessary to drive cell sorting alone is comparatively large (approx. a factor of 10). This suggests that tension asymmetries are the primary driver of cell sorting.

Our results lead us to conclude that, for relatively small MCAs, similar to the sizes of those seen in early development, sorting can occur only if there are relatively large differences in interfacial tension, very large differences in adhesion or both between the different cell types. This could have vast implications in, for example, the early developing embryo where only a few cells are present in an MCA.

Our method can be extended in several ways. For example, one can endow intra-cellular regions with different properties, making it possible to model the nucleus and the plasma membrane. Even more generally, this method can be extended straightforwardly to test aggregates that are not confined to roughly spherical geometries, many of which occur in biological systems. For these reasons, our method can be useful well beyond studying tissue morphogenesis and could be applied to investigate a range of biological processes and pathologies, such as cancer cell metastasis.

## Supplementary Material

Appendices

## Supplementary Material

Simulation movie 1

## Supplementary Material

Simulation movie 2

## Data accessibility

Computational code and accompanying comments can be found at https://github.com/chris-revell/SEM.
