# Supplementary material for: Force-based three-dimensional model predicts mechanical drivers of cell sorting
Source: Proc Biol Sci. 2019 Jan 23;286(1895):20182495. doi: 10.1098/rspb.2018.2495 (PMC6364585; doi:10.1098/rspb.2018.2495)
Supplement: Appendices [file rspb20182495supp1.pdf]

Force-based three-dimensional model predicts mechanical drivers of cell sorting
DOI:10.1098/rspb.2018.2495

Christopher Revell[1], Raphael Blumenfeld[1,3], and Kevin Chalut*[1,2]

[1] *Cavendish Laboratory, Department of Physics, University of Cambridge*
[2] *Wellcome Trust-MRC Cambridge Stem Cell Institute, University of Cambridge*
[3] *Department of Earth Science and Engineering, Imperial College London*

A Subcellular Element Method

A.1 Theoretical Foundations

The subcellular element method was devised as a multi-scale framework for modelling tissue dynamics in three dimensions, incorporating both individual cell structures and sub-cellular features to allow for adaptive shape changes and complex inter- and intra-cell behaviour. The method treats each cell as a cloud of infinitesimal points that interact with their nearest-neighbours via local forces. Thus the behaviour of a cell emerges entirely from the behaviour of underlying elements. Nearest-neighbour interactions occur between elements in the same cell and between elements in different cells, allowing both inter- and intra-cellular behaviour to be modelled with the same mechanism. Figure 1 shows a 2 dimensional diagram of two SCEM cells. Elements are shown as green circles, with dotted black lines showing nearest-neighbour interactions. The two dotted red lines show inter-cell nearest-neighbour interactions.

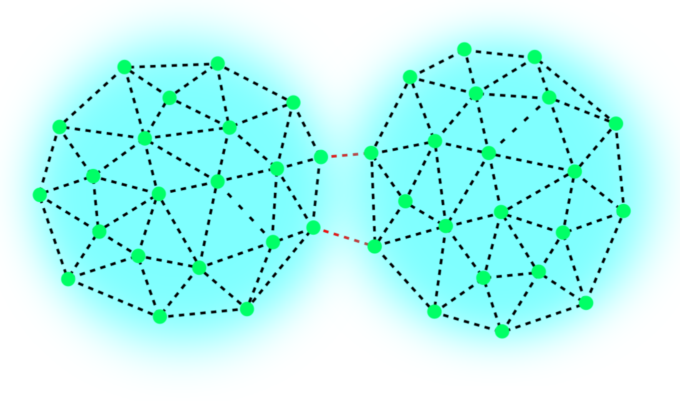

Figure 1: A 2D representation of two SCEM cells, with intra-cellular interactions shown in black, and inter-cellular interactions in red.

The nearest-neighbour interactions between elements take the form of a Morse potential [Equation 1], which is repulsive at short distances, attractive at longer distances and approximately harmonic around

*Corresponding author: kc370@cam.ac.uk

an equilibrium position.

$$V(r) = D_e \left(1 - e^{-a(r-r_e)}\right)^2$$
$$V(r) = D_e \left(e^{-2a(r-r_e)} - 2e^{-a(r-r_e)} + 1\right) \quad (1)$$

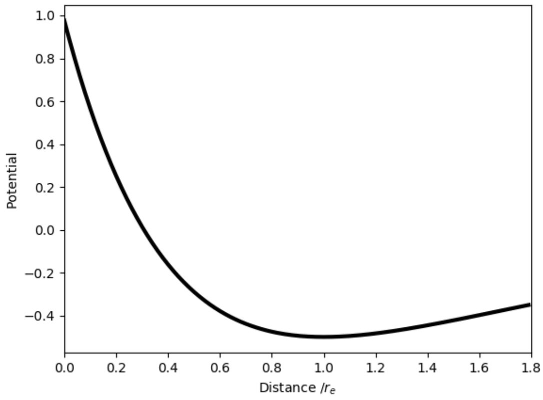

Figure 2: Plot of the basic Morse potential as a function of equilibrium radius.

This potential form has historically been used to model inter-atomic forces in molecules and materials. Figure 2 shows the functional form of the Morse potential for radii between 0 and 1.8 times the equilibrium radius. The threshold separation used for interactions between elements is taken to be 1.8 times the equilibrium radius. Beyond this separation, elements do not interact. Basic values used in simulations are as follows: $r_e = 12.9$, $a = 30.5$, $D_e = 2.29$. The equilibrium length scale for interactions is derived from the typical cell length scale provided as a parameter of the model, the value of which is outlined in Appendix B. Magnitudes of interactions are varied in the acquisition of results described in this paper.

The mechanical properties of a single cell arise from the nearest-neighbour interactions between internal elements. These properties have not been altered from the original published SCEM technique. The mechanical properties of cells arising from this technique were investigated in Sandersius and Newman 2008 [30]. This study employed virtual rheology studies of SCEM cells to demonstrate that the viscoelastic properties of SCEM cells were consistent with those of living cells.

For our purposes, it is relevant to note that the Morse potential can be broken into an attractive and a repulsive component. For inter-cellular interactions other than cortex element to cortex element interactions, the adhesive component of the potential was removed, so $V(r) = D_e \left(e^{-2a(r-r_e)} + 1\right)$. This prevents cells from overlapping in space. Additionally, the inter-cellular adhesive component was increased between cortex elements in order to compensate for the reduction in cell size arising from the introduced cortical tension, so $V(r) = D_e \left(e^{-2a(r-r_e)} - 0.8e^{-a(r-r_e)} + 1\right)$. The gradient of this potential gives the adhesion force, but since this varies with distance, the adhesion magnitude, A_M, was defined as the maximum value of this adhesive force over the interaction range; in other words, the steepest slope of the potential. The magnitude of the adhesion was varied between simulations by multiplying the potential by a user-specified value.

At each time step of a simulation, element positions are updated according to the forces acting upon them using overdamped Langevin dynamics, as shown in Eq. 2, where γ is a drag factor, U is the potential, $\underline{x}$ is the element position, and $\underline{\zeta}$ is a stochastic component. Values for parameters such as drag factor have not been updated from the original SCEM implementation, nor has the interaction cut off at 1.8 times the equilibrium radius.

$$\gamma \dot{\underline{x}} = -\nabla U(\underline{x}) + \underline{\zeta} \quad (2)$$

The stochastic term in Eq. 2 is implemented as an update to the element position, using a random number generator to determine direction and with the distance chosen at random from a Gaussian

distribution. The properties of this Gaussian distribution are set by parameters described in Appendix B.

A.2 Modifications From the Original SCEM

The original SCEM code considered each cell as a uniform entity. Notably the primary modification we made was to add cortex elements with tensile forces that act within the elements of the cortex and with other cells' cortices. The addition of these cortices allowed us to control intercellular adhesion and tension independently in order to comprehensively test the differential interfacial tension mechanism of cell sorting.

A.3 Growth and Division

Cells in SCEM are able to grow from their initial size to double their initial volume. This is achieved by adding new elements to the centre of each cell and allowing the cell to relax in response to the new forces between introduced and existing elements. The creation of a new element object at the centre of a cell introduces new nearest-neighbour interactions with existing elements, forcing them apart and increasing the cell volume. New elements are introduced at a random position within a limited radius from the cell centre of mass. This limit is 90% of the cell's radius of gyration.

The average rate at which elements are introduced to the centre of the cell is controlled by the cell cycle time. The probability of adding a new element at each time step is inversely proportional to the cell cycle time such that the number of elements in a given cell is doubled within approximately the cell cycle time: `prob_new_element=ne_cell*dt/cell_cycle_time`. This gives the rate of growth an inherent randomness but with a characteristic average rate. The value of the cell cycle time is given in Appendix B.

Once a cell reaches double the initial number of elements, division is triggered. The SCEM division algorithm finds the longest axis of a cell by testing all possible sets of two elements within the cell until the two with the greatest separation are found, and then bisects the cell perpendicular to this axis, allocating elements on either side of the division plane to the two newly defined daughter cells. In our updated version, cortex element allocation is performed after division to introduce new cortex at the boundary between the two daughter cells. Since cell division depends on cell size, and cell growth has an inherent randomness, division is not synchronised but has a characteristic timescale set by the cell cycle time.

A.4 Simulation Protocol

For the purposes of the results shown in this paper, simulations began with a single cell created *de novo* from 128 elements. This cell was allowed to grow and divide until an aggregate of 10 identical cells was formed. At this point cells in the system were randomly allocated different cell types. Any random allocation of cell types that did not produce equal numbers of the two cell types (or a difference of 1, for an odd number of cells) is rejected and the random type allocation is repeated until this criterion is met. Once two cell types have been allocated, physical differences between the two types were introduced. At this point we began calculating and recording sorting measures, and the simulation continued until the aggregate contained 30 cells, signalling the end of the simulation.

A_M is a property of the Morse potential governing the interactions between elements, and is implemented by varying the prefactor of inter-cell adhesive interactions to change the magnitude of forces between elements. γ_m is implemented by varying the contant force acting between neighbouring cortex elements as defined by the Delaunay triangulation. γ_c is implemented by locally multiplying the value of γ_m by a factor β.

B Simulation Parameters

A breakdown of the parameters of interest for our system is shown below. The ranges of each parameter used in our investigations are given in the paper.

Variable	Description
A_M	Peak adhesive force produced by potential between elements in different cells.
γ_m	Tension force acting between neighbouring cortex elements within a cell.
γ_c	Tension force acting between neighbouring cortex elements within a cell at a cell-cell interface.
β	Factor by which cortical tension is varied at cell-cell interfaces ($= \gamma_c/\gamma_m$).
α	Dimensionless adhesion parameter defined by adhesion magnitude divided by cortical tension ($= A_M/\gamma_m$).

Further parameters of original SCEM model [24] not explicitly investigated nor varied in this work are outlined below.

Variable	Description
`viscous_timescale_cell`	= 100 Time scale of viscous relaxation for cell. Combined with `elastic_mod_cell`, determines drag factor of elements in Langevin equation.
`elastic_mod_cell`	= 1.0 Elastic modulus. Defines effective spring constant of near-equilibrium Morse potential.
`r_cell`	= 10.0 Typical cell lengthscale. Defines Morse potential interaction radius.
`cell_cycle_time`	= 1500 Cell cycle time. Defines cell growth rate.
`dt_amp_max`	= 0.1 Used to calculate `dt`, the time interval between simulation steps. `dt` α `dt_amp_max`/Maximum force magnitude
`frac_interaction_max`	= 1.8 Limit of Morse potential interaction radius relative to equilibrium radius.
`epsilon`	= 0.01 Value of Morse potential relative to minimum at maximum interaction radius.
`stiffness_factor`	= 1.0 Scaling factor for internal cytoplasm interactions.
`diff_coeff`	= 0.001 Diffusion coefficient of elements. Used to calculate stochastic term.

C Cell Cortex Smoothness

The cell cortex is defined by a Delaunay triangulation over cortex elements as explained in the main text of the paper. We require a smooth cell surface, and confirmed that this was produced by testing

the distribution of edges per vertex in the triangulation. Significant spread in this distribution would suggest variation between cortex elements, and consequently not a smooth cell surface. The normalised frequency density distribution of this edge count for a single cell is shown in Figure 3. The minimum number of interactions experienced by any cortex element is 3, and the maximum is 16, but there exists a sharp peak in the distribution, with roughly 65% of all cortex elements experiencing 6 interactions. This sharp peak in the distribution indicates a uniform smoothness over the cell surface.

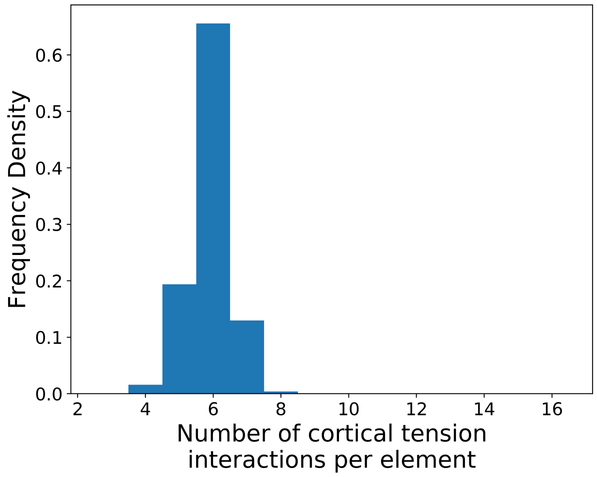

Figure 3: Frequency density distribution of the number of cortical tension interactions per cortex element in a single cell. The sharp peak around 6 indicates a relative uniformity across the cell surface.

D Decoupling Tension From Adhesion

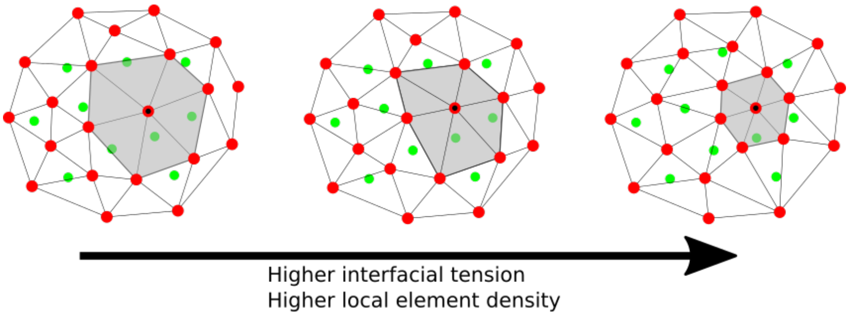

Figure 4: Diagram demonstrating how local area around an element is calculated to produce a normalisation factor that decouples the local adhesion magnitude from the local element density, and hence from the local cortical tension magnitude. The inter-cell adhesion magnitude of the element labelled with a black dot is multiplied by the grey-shaded area defined by all Delaunay triangles that have the element with a black dot as a vertex, divided by the mean such area found for isolated cells in equilibrium. Thus the magnitude of adhesion is inversely proportional to the local density of elements.

Care is required when implementing the DIT algorithm described in this paper. Changes to the local cortical tension magnitude in cortex-cortex interaction pairs will change the distance between elements in the pairs and thus result in changes to the local density of elements. Since adhesion between cells is mediated by these elements, a change in the element density will affect the local adhesion strength between neighbouring cells. This could counteract the expected effects of differential interfacial tension. For example, increasing the local tension at an interface, which should reduce the affinity between two cells, will result in a higher density of elements and thus a stronger local adhesion between the cells, potentially increasing their affinity in opposition to the effect of the change in tension.

To solve this problem, we devised an algorithm to normalise the adhesion magnitude of an element by the local element density. We begin by calculating the total area of all triangles in the Delaunay triangulation of cortex elements that have the element under consideration as one of their vertices

[Figure 4]. We then divide this area by the mean area for elements in a single cell at equilibrium to find a factor by which the area has changed relative to equilibrium. This factor is then used to change the magnitude of the adhesive interactions of the element. Thus any change from the equilibrium area will produce a corresponding change in local adhesion magnitude.

E Cell State Reallocation Repetitions

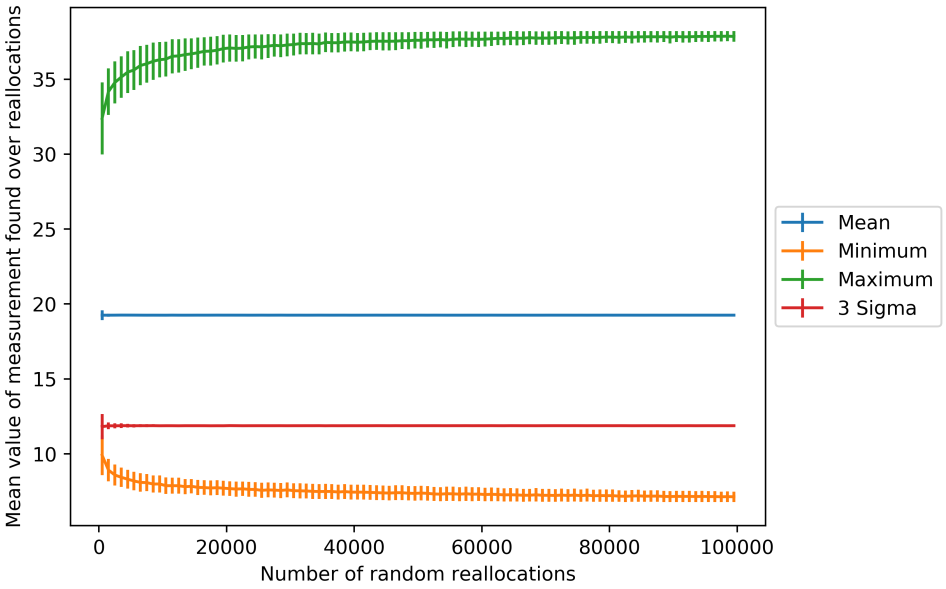

Figure 5: Variation of mean, maximum, minimum, and standard deviation in neighbour measure against the number of random orientations tested in a system of 30 cells. The values of these variables are found with small errors for many fewer random tests than the total possible number of reallocations.

The distribution of values from randomised sorting measures is approximately Gaussian, allowing us to calculate a mean, standard deviation, maximum, and minimum value. Before performing the randomised measurement routine it's important to establish how many random reallocations to perform. A 30 cell system with 15 cells of each type has almost 1.6×10^8 possible different arrangements, and it is impossible to sample all such systems without increasing the run time of a simulation beyond what is computationally feasible. Fortunately, we were able to show that values quickly tend towards a limit over a much smaller number of repetitions. Figure 5 shows how the mean, standard deviation, maximum, and minimum values found across random reallocations vary with the number of random reallocations tested for typical systems of 30 cells. It can be seen that the mean and standard deviation values are found with very little error over fairly small numbers of reallocations, whilst the maximum and minimum values are found to high accuracy on average after about 10^4 reallocations. Thus we chose to use 10^4 reallocations in our simulations. The exception to this is for small systems in which the total number of possible orientations is smaller than 10^4, in which case the maximum is used instead.

F Supplementary Movies Summary

Two movies accompany this paper. Both are animated visualisations created using POV-Ray, and visualising the Delaunay triangulation at the boundary of each cell as a continuous surface. Each movie shows a simulation running from 10 cells to 30 cells, with cell type 1 coloured green and cell type 2 coloured red. Simulations are run under the same conditions as the main results for this paper. Cell type 2 has no change in its interfacial tension at any interfaces in both movies. In Movie 1, cell type 1 has $\beta = 0.5$ for like-like interfaces, and thus sorts to the inside of the aggregate. In Movie 2, cell type 1 has $\beta = 1.0$ for like-like interfaces, and thus no sorting occurs.
