# Supplementary figures and images for: Force-based three-dimensional model predicts mechanical drivers of cell sorting
Source: Proc Biol Sci. 2019 Jan 23;286(1895):20182495. doi: 10.1098/rspb.2018.2495 (PMC6364585; doi:10.1098/rspb.2018.2495)

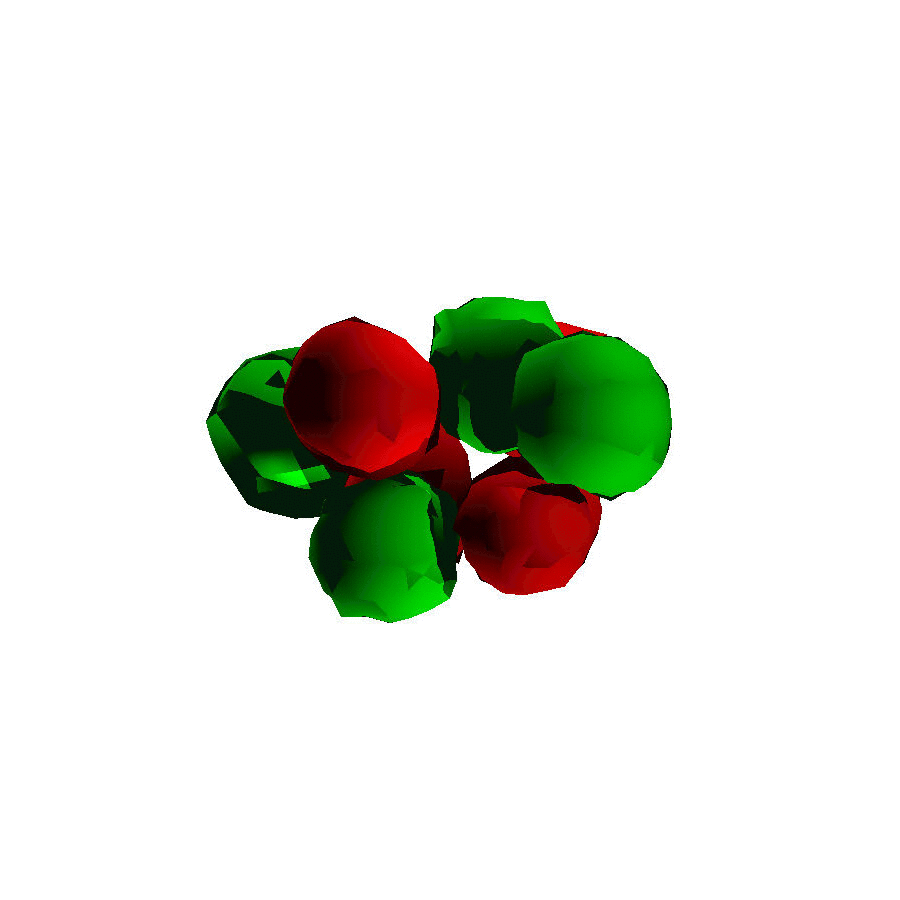

Supplement: Simulation movie 1 [file rspb20182495supp2.gif]

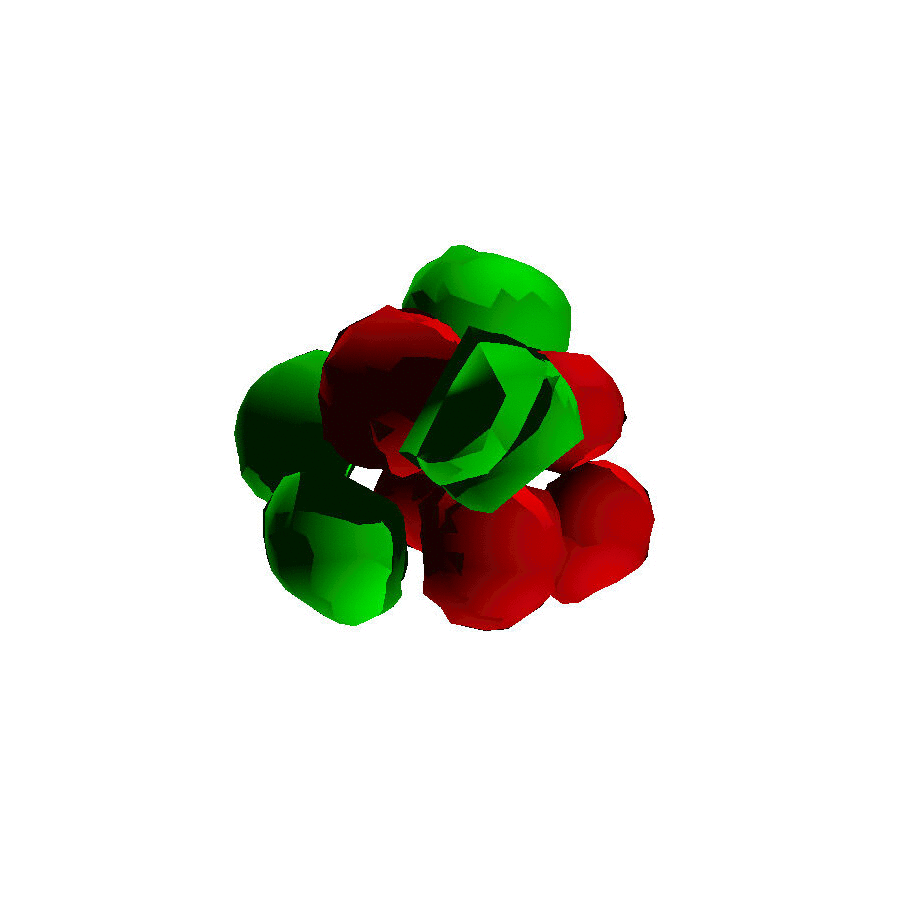

Supplement: Simulation movie 2 [file rspb20182495supp3.gif]
